# Autophagy Receptor Tollip Facilitates Bacterial Autophagy by Recruiting Galectin-7 in Response to Group A *Streptococcus* Infection

**DOI:** 10.3389/fcimb.2020.583137

**Published:** 2020-12-23

**Authors:** Ching-Yu Lin, Takashi Nozawa, Atsuko Minowa-Nozawa, Hirotaka Toh, Miyako Hikichi, Junpei Iibushi, Ichiro Nakagawa

**Affiliations:** Department of Microbiology, Graduate School of Medicine, Kyoto University, Kyoto, Japan

**Keywords:** tollip, autophagy, group A *Streptoccocus*, galectin 7, galectin 1

## Abstract

Bacterial autophagy—a type of macroautophagy that is also termed xenophagy—selectively targets intracellular bacteria such as group A *Streptococcus* (GAS), a ubiquitous pathogen that causes numerous serious diseases, including pharyngitis, skin infections, and invasive life-threatening infections. Although bacterial autophagy is known to eliminate invading bacteria *via* the action of autophagy receptors, the underlying mechanism remains unclear. Herein, we elucidated that Tollip functions as a bacterial-autophagy receptor in addition to participating involved in the intracellular immunity mechanism that defends against bacterial infection. Tollip was recruited to GAS-containing endosomal vacuoles prior to the escape of GAS into the cytosol; additionally, Tollip knockout disrupted the recruitment of other autophagy receptors, such as NBR1, TAX1BP1, and NDP52, to GAS-containing autophagosomes and led to prolonged intracellular survival of GAS. Furthermore, Tollip was found to be required for the recruitment of galectin-1 and -7 to GAS-containing autophagosomes, and immunoprecipitation results indicated that Tollip interacts with galectin-7. Lastly, our data also revealed that galectin-1 and -7 are involved in the restriction of GAS replication in cells. These results demonstrated that Tollip modulates bacterial autophagy by recruiting other autophagy receptors and galectins.

## Introduction

Autophagy is a highly conserved intracellular metabolism mechanism in which autophagosome -lysosome fusion facilitates the clearance of intracellular components such as protein aggregates, damaged organelles, and invading pathogens (Ohsumi, 2013). Selective autophagy of pathogens, also termed xenophagy, specifically targets intracellular pathogens such as bacteria and viruses for clearance through the fusion of pathogen-containing autophagosomes with lysosomes (Nakagawa et al., 2004; Moy et al., 2014). The selective autophagy of pathogens in the cytoplasm is mediated by autophagy receptors that connect their targets to autophagic membranes. To date, p62/SQSTM1, NDP52, OPTN, and TAX1BP1 have been reported to function as autophagy receptors in bacterial autophagy (Okamoto, 2014). Group A *Streptococcus* (GAS) is a major human pathogen that caused numerous serious diseases (Cole et al., 2011). It has been reported previously that autophagy functions as a crucial intracellular immune mechanism in the defense against GAS invasion (Nakagawa et al., 2004; Tumbarello et al., 2015; Franco et al., 2017).

During GAS infection, internalized GAS escapes from endosomes into the cytosol by secreting the pore-forming toxin streptolysin O (SLO); it is subsequently captured by autophagosomes, which fuse with lysosomes for bacterial degradation. Cytosolic bacteria are first demarcated by ubiquitin binding. Thereafter, ubiquitin-binding autophagy receptors such as p62/SQSTM1 and NDP52 are recruited to the ubiquitin-coated bacteria, and these receptors then drive autophagosome formation around the invading bacteria (Ito et al., 2013; Minowa-Nozawa et al., 2017). Moreover, we recently reported that TAX1BP1 is involved in the fusion of GAS-containing autophagosomes with lysosomes (Lin et al., 2019). Although the detailed mechanisms of action and the functions of autophagy receptors remain incompletely elucidated, they are recognized to be fundamentally critical for GAS autophagy because they regulate its various steps. Also, clarifying which autophagy receptors are involved in bacterial autophagy is crucial to understanding the autophagy machinery that functions in response to bacterial infection.

Apart from ubiquitin-mediated bacterial recognition, a mechanism involving galectins mediates the detection of cell-invading bacteria; intracellular galectins serve as bacterial sensors that are recruited to the damaged membrane debris surrounding cytosolic bacteria, and this is followed by the cellular autophagy response for bacterial clearance (Thurston et al., 2012; Chen et al., 2014). Among the galectin-family proteins, galectin-3, -8, and -9 have been demonstrated to be recruited to bacterium-containing vesicles and to function in autophagy induction (Thurston et al., 2012; Jia et al., 2018). Galectin-3 guides the recruitment of ATG16L1 to the damaged endosomal membrane and triggers the autophagy response (Chauhan et al., 2016), and galectin-8 is required for the recruitment of NDP52 during *Salmonella enterica serovar Typhimurium* infection; conversely, galectin-3 is reported to block the recruitment of galectin-8 and E3 ligase to bacteria in GAS-infected endothelial cells (Cheng et al., 2017).

Herein, we focused on the protein Tollip, a human homolog of the yeast ubiquitin-Atg8 adaptor Cue5. Tollip is reported to function as a ubiquitin-LC3 adaptor in the autophagy pathway (Lu et al., 2014), but whether it is involved in bacterial autophagy remains unknown. The present study delineates distinct functions of Tollip in regulating the recruitment of galectin-1 and -7 to bacterium-containing vesicles. It was also demonstrated herein that galectin-1 and -7 are pivotally involved in the intracellular immunity mechanism that defends against GAS infection.

## Materials and Methods

### Antibodies

The following primary and secondary antibodies were used in the study: mouse monoclonal anti-galectin-3 (1:250, B2C10; BD Biosciences, 556904), rabbit polyclonal anti-galectin-7 (1:250 dilution for immunofluorescence, 1:1000 dilution for Western blot, Abcam, ab10482), mouse monoclonal anti-LAMP-1 (1:250, H4A3; Santa Cruz Biotechnology, sc-20011), rabbit polyclonal anti-Tollip (1:1000, Abcam, ab187198), rabbit monoclonal anti-NBR1 (1;250, D2E6; Cell Signaling, 9891), mouse monoclonal anti-p62 (1:250, D-3; Santa Cruz Biotechnology, sc-28359), rabbit monoclonal anti-TAX1BP1 (1:250, D1D5; Cell Signaling, 5105), mouse monoclonal anti-streptolysin (1:2000, 6D11; Abcam, ab23501), mouse monoclonal anti-GFP (1:1000, GF200; Nacalai Tesque, 04363‐24), mouse monoclonal anti-GAPDH (1:1000, 6C5; Santa Cruz Biotechnology, sc-32233), and mouse monoclonal anti-FLAG (1:1000, M2; Sigma-Aldrich, A2220). The secondary antibodies used were the following: for immunoblotting, HRP-conjugated anti-rabbit and anti-mouse IgG (1:4000, Jackson ImmunoResearch Laboratories); for immunostaining, anti-mouse or anti-rabbit IgG conjugated with AlexaFluor-488/-594 (1:250, Molecular Probes/Invitrogen).

### Plasmids

The generation of Human Galectin-3, Galectin-8, and LC3 has been described previously (Ankem et al., 2011). Human Tollip, Galectin-1, Galectin-2, and Galectin-7 were amplified by PCR from total mRNA derived from HeLa, KYSE, and HEK293T cells and cloned into pcDNA-6.2/N-EmGFP-DEST, pcDNA-6.2/N-3xFLAG-DEST, and pcDNA-6.2/N-mCherry-DEST using Gateway technology (Invitrogen) as previously described (Toh et al., 2019).

### Bacterial Strains

GAS strain JRS4 (M6+ F1+), SLO-deletion-mutant GAS (Toh et al., 2019), and *S. aureus* KUH180129 (Hikichi et al., 2019) were grown in Todd-Hewitt broth (BD Diagnostic Systems, 249240) supplemented with 0.2% yeast extract.

### Cell Culture

HeLa cells and HaCaT cells were cultured in DMEM containing 10% fetal bovine serum (Gibco) and 50 μg/mL gentamicin (Nacalai Tesque).

### CRISPR/Cas9-Mediated Gene Editing and siRNA-Mediated Gene Knockdown

The CRISPR/Cas9 gene-editing system was used to generate Tollip-knockout HeLa cells and galectin-1-knockout HeLa cells (Mali et al., 2013), following the protocol described previously (Oda et al., 2016). The guide RNA (gRNA) sequences were 5‘-ACCACCGTCAGCACTCAGCG-3‘ for Tollip, 5‘-GACCCTCGCTTCAACGCCCA-3‘ for Galectin-1 knockout. For knockdown experiments, cells were transfected with Galectin-7 siRNA oligonucleotides (s198075; Thermo Fisher Scientific), ATG5 siRNA oligonucleotides (s18160; Thermo Fisher Scientific), or nontargeting siRNAs (12935300, Thermo Fisher Scientific) using Lipofectamine 3000 (Invitrogen). Knockdown was confirmed by immunoblotting.

### Immunofluorescence

HeLa cells were seeded and grown on coverslips in 24-well plates. After removing the culture medium, cells were fixed and permeabilized by treating them sequentially with 4% paraformaldehyde for 15 min at 25°Cand 0.1% Triton X-100 in PBS for 10 min. Thereafter, cells were blocked using PBS containing 2% BSA and 0.02% sodium azide. To label target proteins, cells were incubated with blocking buffer containing primary antibodies for 1 h at room temperature, washed thrice with PBS, and then incubated with the appropriate secondary antibodies conjugated with Alexa Fluor-488 or -594 for 1 h at room temperature.

### Immunoprecipitation and Immunoblotting

HeLa cells were transfected with EmGFP-galectin-7 and FLAG-Tollip by using polyethylenimine (Polysciences) reagent, and the transfected cells were lysed using a buffer containing 10 mM Tris-HCl, pH 7.4, 1% NP40, 150 mM NaCl, 1 mM EDTA, and 5% glycerol. The lysates were centrifuged at 15,000 x g for 20 min at 4°C. The supernatants were transferred to new Eppendorf tubes and incubated with 2 μl of anti-FLAG M2 antibody at 4°C for 2 h and then with Protein G Sepharose 4B (GE Healthcare Life Sciences) at 4°C for 1 h. Lastly, the beads were washed five times with the cell-lysis buffer at 4°C and then heated with 2× Laemmli sample buffer at 95°C for 5 min to obtain the immunoprecipitates. For immunoblotting, proteins were separated using SDS-PAGE, transferred to PVDF membranes, and detected using specific primary antibodies (1 h, room temperature) and HRP-conjugated secondary antibodies.

### Bacterial-Invasion Assay

Bacterial infection was assayed using the protocol described previously (Lin et al., 2019). Briefly, HeLa or HaCaT cells were seeded in 24-well plates at a density of 4.5 × 10^4^ cells/well, washed twice with 1× PBS, and infected with GAS at a multiplicity of infection of 100 for 1 h in DMEM without antibiotics. After washing twice more with 1× PBS, the cells were cultured in medium containing 100 μg/mL gentamicin to eliminate the bacteria that had not been internalized by the cells. The GAS-infected cells were then harvested at different times (as indicated in the figures).

### Statistical Analysis

Colocalization and GAS-containing autophagosome formation were quantified through direct visualization performed using confocal microscopy. Unless indicated otherwise, at least 50 autophagosomes or 200 GAS-infected cells were counted per condition in each experiment, and at least three independent experiments were performed for each trial. The values, including those displayed in the graphs, represent mean ± SD. Statistical analysis was performed by two-tailed Student’s *t*-tests. *P* values less than 0.05 were considered to indicate statistical significance, and are marked * for *P* < 0.05, ** for *P* < 0.01.

## Results

### Tollip Is Localized to GAS-Containing Vacuoles and Colocalized ith LC3

**Figure 1:**
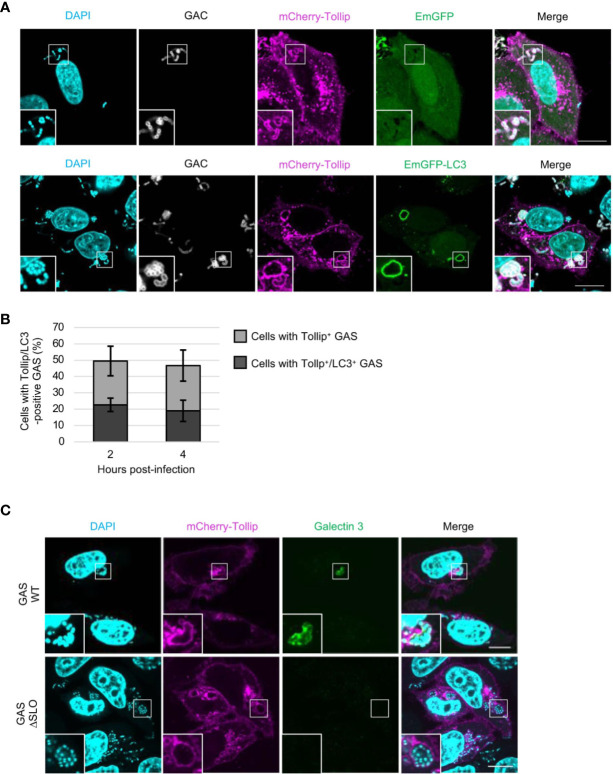
Tollip is recruited to GAS-containing vacuoles before GAS damages the endosomal membrane. **(A)** HeLa cells transfected with mCherry-Tollip and EmGFP-LC3 were infected with Group A *Streptococcus* (GAS) for 4 h, and immunostained with anti-Group A *Streptococcus* carbohydrate (GAC) to stain GAS. Immunofluorescence analysis was performed to determine the localization of mCherry-Tollip and EmGFP-LC3. Cellular and bacterial DNA were stained with DAPI. Insets: enlarged boxed areas. All images are representative of at least 3 independent experiments. Scale bar, 10 μm. **(B)** Percentages of cells containing Tollip-positive GAS and cells containing Tollip-positive autophagosomes; cells were manually counted using immunofluorescence confocal microscopy. In each experiment, >60 autophagosomes were evaluated per sample; bars: means ± SD of 3 independent experiments. **(C)** HeLa cells were transfected with mCherry-Tollip and infected with GAS or SLO-deletion-mutant (ΔSLO) GAS for 4 h, and then stained for endogenous galectin-3. Immunofluorescence microscopy was used to examine the localization of mCherry-Tollip and endogenous galectin-3. Insets: enlarged boxed areas. All images are representative of at least 3 independent experiments. Scale bar, 10 μm.

To determine whether Tollip is involved in the xenophagy triggered in response to bacterial infection, we first evaluated the localization of Tollip in GAS-infected HeLa cells. HeLa cells expressing mCherry-Tollip and EmGFP-LC3, a marker of autophagic membranes, were infected with GAS, and stained with anti-Group A *Streptococcus* carbohydrate (GAC) antibody and DAPI. DAPI signals other than cellular nuclei completely matched with GAC (**Figure S1A**), and intracellular GAS were surrounded by mCherry-Tollip (**Figure 1A**). GAS-surrounding Tollip was also colocalized with EmGFP-LC3 (**Figure 1A**). Approximately 50% of the cells contained mCherry-Tollip-positive GAS and nearly 20% harbored mCherry-Tollip-positive GAS-containing autophagic vacuoles at 2 and 4 h post-infection (**Figure 1B**); this implied that LC3 is recruited to Tollip-positive GAS.

Next, to ascertain whether Tollip is recruited to GAS owing to the endomembrane damage caused by GAS for its escape into the cytosol, cells were infected with SLO-deletion-mutant GAS (ΔSLO), which lacks pore-forming ability. Thereafter, staining with galectin-3, a marker of damaged vacuole membranes, was conducted to investigate the extent of endomembrane damage caused by SLO protein. The obtained results, suitably depicted in images, showed that mCherry-Tollip was recruited to, and surrounded, both galectin-3–positive and galectin-3–negative GAS (**Figure 1C**). This, in turn, signified that Tollip was recruited to GAS in a SLO-independent manner. This result is concordant with the findings of a previous study that Tollip is localized to endosome membranes (Mitra et al., 2013).

To examine whether the GAS contained within Tollip-positive endosomes disrupts the membrane to escape into the cytosol, we examined the localization of EmGFP-tagged galectins. It was ascertained that galectin-3 and -8, as well as galectin-1 and -7, colocalized with GAS-surrounding Tollip at 4 h post-infection (**Figures S1B, C**). We further determined that mCherry-Tollip was colocalized with galectin-1-, -3-, -7-, or -8-positive *Staphylococcus aureus*, a major gram-positive bacterial pathogen (**Figure S2A**). In addition, galectin-7 was recruited to, and colocalized with, LC3-surrounded *S. aureus* (**Figure S2B**). These results indicated that galectin-1 and -7, similar to galectin-3 and -8, were accumulated on the bacterial surface during infection.

### Tollip Is Involved in Xenophagy Against Group A *Streptococcus*

**Figure 2:**
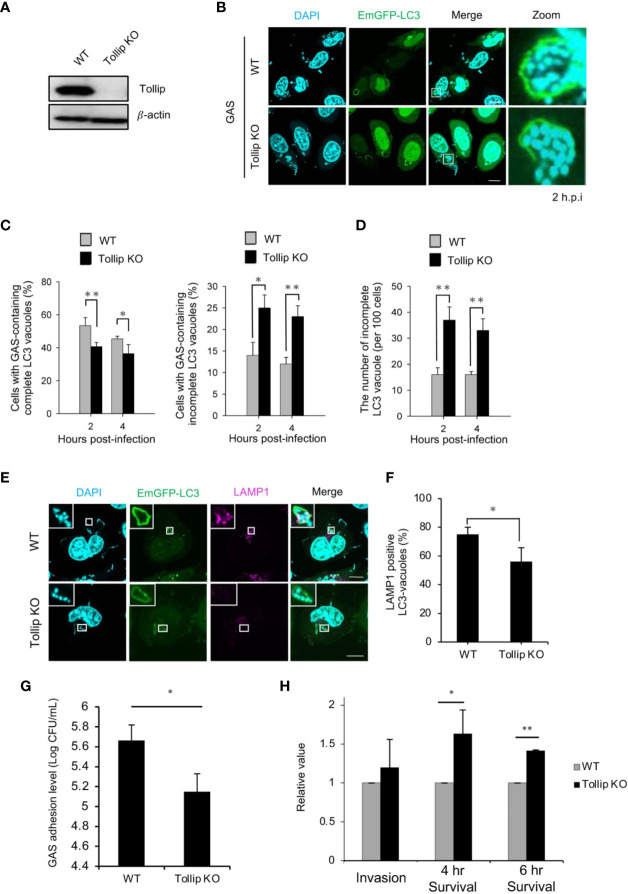
Tollip is required for autophagosome and autophagolysosome formation during GAS infection. **(A)** Tollip-knockout (KO) cells were generated using CRISPR/Cas9-mediated gene editing. Immunoblotting was used to detect Tollip protein expression in wild-type (WT) and Tollip-knockout cells. **(B)** HeLa cells transfected with EmGFP-LC3 were infected with GAS, and at 2 hpi, immunofluorescence analysis was performed to examine the formation of bacterium-containing autophagosomes. **(C)** Cells harboring GAS-containing autophagosomes (left) and cells containing LC3-positive fragments (right) were manually counted using immunofluorescence confocal microscopy. In each experiment, >100 autophagosomes were evaluated per sample; bars: means ± SD of 3 independent experiments. *P* values less than 0.05 were considered to indicate statistical significance, and are marked * for *P* < 0.05, ** for *P* < 0.01. **(D)** LC3-positive fragments in cells were manually counted and examined using confocal microscopy. In each experiment, 100 LC3-positive cells were evaluated per sample; bars: means ± SD of 3 independent experiments. **(E)** HeLa cells transfected with EmGFP-LC3 were infected with GAS, and at 4 hpi, immunofluorescence analysis was used to examine the localization of EmGFP-LC3 and endogenous LAMP1. Insets: enlarged boxed areas. All images are representative of at least 3 independent experiments. Scale bar, 10 μm. **(F)** LAMP1-positive GAS-containing autophagosomes were manually counted using immunofluorescence confocal microscopy. In each experiment, 100 LC3-positive cells were evaluated per sample; bars: means ± SD of 3 independent experiments. **(G)** The adhesion level of GAS in wild-type and Tollip-knockout HeLa cells. **(H)** Invasion rate and survival rate of GAS in wild-type and Tollip-knockout HeLa cells.

To determine whether a loss of Tollip function affects bacterial autophagy, we generated Tollip-knockout HeLa cells by employing CRISPR/Cas9-mediated gene editing (**Figure 2A**). The resulting knockout cells were infected with GAS, and the autophagic vacuoles were examined by labeling with EmGFP-LC3 and performing confocal microscopy. In wild-type cells, LC3 signals completely enclosed intracellular GAS in a circular pattern, whereas in Tollip-knockout cells, the LC3-positive vacuoles surrounding GAS were incomplete and not closed (**Figure 2B** and **Figure S3**). Manual counting of cells harboring complete and incomplete LC3 vacuoles among the wild-type and Tollip-knockout populations (**Figure 2C**) revealed that completely detectable autophagosomes were markedly decreased in Tollip-knockout cells. However, there was an increase in the percentage of cells harboring incomplete LC3 vacuoles, and the number of incomplete LC3 vacuoles was also drastically elevated in Tollip-knockout cells (**Figures 2C, D**). These results signified that Tollip is involved in autophagosome formation during GAS infection.

Because autophagosomes fuse with lysosomes after the autophagic membrane completely encloses the target, we examined the lysosomal fusion of LC3-positive membranes in Tollip-knockout cells. The rate of recruitment of LAMP1, a lysosome marker, to LC3-vacuoles was significantly decreased in Tollip-knockout cells relative to that in wild-type cells (**Figures 2E, F**), which indicated that autophagolysosome formation was suppressed following Tollip knockout. We next examined whether GAS adhesion ability was affected in Tollip knockout cells. The result indicated that GAS adhesion level was significantly decreased in Tollip knockout cells (**Figure 2G**). Furthermore, we examined whether the loss of Tollip affects bacterial degradation through autophagy, and we found that the proliferation rate of GAS was markedly increased in Tollip-knockout cells (**Figure 2H**). Collectively, our results indicated that Tollip plays a critical role in the formation of autophagosomes and autophagolysosomes for the elimination of invading GAS.

### Tollip Is Required for NBR1, TAX1BP1, and NDP52 Recruitment

**Figure 3:**
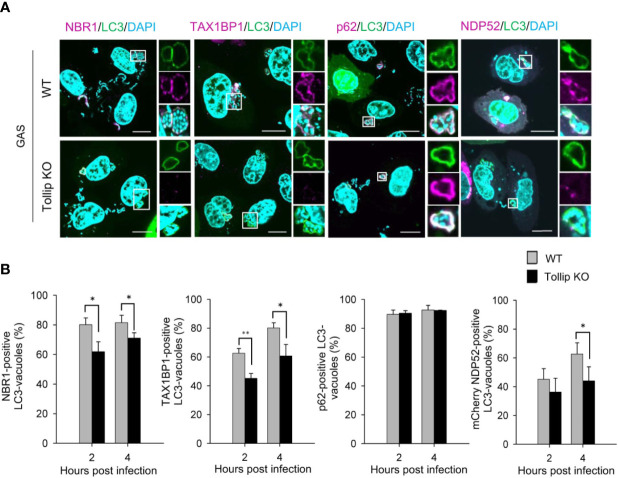
Tollip is required for the recruitment of NBR1, TAX1BP1, and NDP52 to GAS-containing LC3 vacuoles. **(A)** Wild-type and Tollip-knockout HeLa cells were transfected with EmGFP-LC3 or co-transfected with EmGFP-LC3 and mCherry-NDP52. After infecting with GAS for 4 h, endogenous NBR1, TAX1BP1, and p62 were labeled with antibodies. Insets: enlarged boxed areas; top: EmGFP-LC3; middle: autophagy receptors; bottom: merged image. All images are representative of 3 independent experiments. Scale bar, 10 μm. **(B)** Percentages of recruitment of autophagy receptors to GAS-containing autophagosome-like vacuoles: NBR1, TAX1BP1, p62, and mCherry-NDP52 (from left to right). In each experiment, >50 LC3-positive vacuoles were evaluated per sample; bars: means ± SD of 3 independent experiments. *P < 0.02, **P < 0.01.

Because Tollip mediates the recruitment of autophagy receptors to protein aggregates during aggrephagy (Lu et al., 2014), we next investigated whether the recruitment of autophagy receptors to GAS-containing vacuoles is also impaired in Tollip-knockout cells. Loss of Tollip perturbed the recruitment of NBR1, TAX1BP1, and NDP52 to GAS-containing LC3-vacuoles (**Figure 3A**); additionally, the proportion of NBR1-, TAX1BP1-, and NDP52-positive GAS-containing LC3-vacuoles was significantly decreased in Tollip-knockout cells (**Figure 3B**). These results implied that Tollip functions in the recruitment of NBR1, TAX1BP1, and NDP52 in xenophagy.

### Galectin-1 and -7 Target Invading Group A *Streptococcus via* Tollip

**Figure 4:**
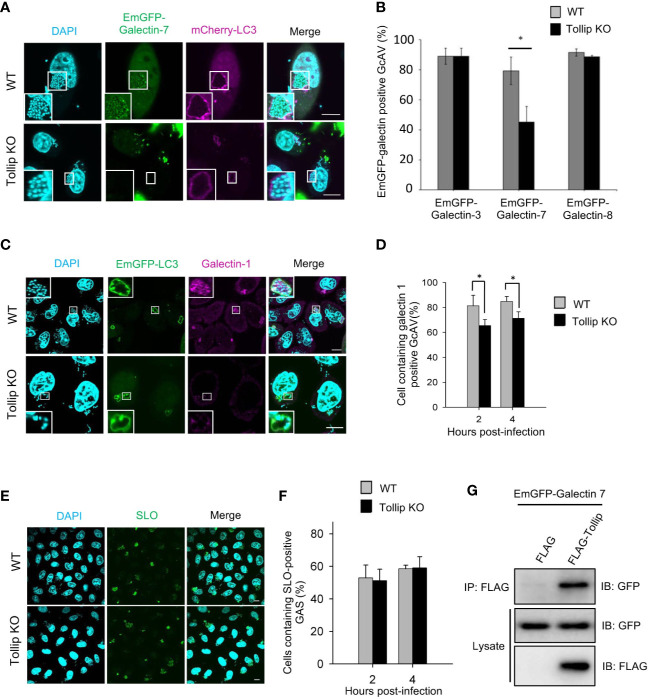
Tollip is involved in the recruitment of galectin-1 and -7 to GAS-containing LC3-vacuoles. **(A)** Wild-type and Tollip-knockout HeLa cells transfected with EmGFP-galectin-7 and mCherry-LC3 were infected with GAS; at 4 hpi, immunofluorescence analysis was performed to examine the localization of the expressed proteins. **(B)** EmGFP-galectin-positive autophagosomes were manually counted using immunofluorescence confocal microscopy. In each experiment, >50 GAS-containing LC3-vacuoles were counted and evaluated per sample; bars: means ± SD of 3 independent experiments. **(C)** Wild-type and Tollip-knockout HeLa cells transfected with EmGFP-LC3 were infected with GAS; at 4 hpi, immunofluorescence analysis was used to examine the localization of endogenous galectin-1 and EmGFP-LC3; galectin-1 was labeled using a rabbit anti-galectin-1 antibody. **(D)** Cells containing galectin-1-positive GAS-containing autophagosomes were manually counted using immunofluorescence confocal microscopy. In each experiment, >50 GAS-containing autophagosomes were counted and evaluated per sample; bars: means ± SD of 3 independent experiments. **(E)** Wild-type and Tollip-knockout HeLa cells were infected with GAS; at 4 hpi, immunofluorescence analysis was performed to examine the SLO protein expression from GAS. Scale bar, 10 μm. **(F)** Cells containing SLO-positive GAS were manually counted using immunofluorescence confocal microscopy. In each experiment, >200 GAS-infected cells were evaluated per sample; bars: means ± SD of 3 independent experiments. **(G)** Tollip interacts with galectin-7. HeLa cells were co-transfected with EmGFP-galectin-7 and FLAG-Tollip for 48 h, after which immunoprecipitation was performed using anti-FLAG. The immunoprecipitates were immunoblotted with anti-GFP to detect EmGFP-galectin-7. Experiments were repeated more than three times. *P < 0.05.

Galectins are recognized to function as a link between the damaged endomembrane and the autophagy receptor NDP52 or core autophagy regulators (Thurston et al., 2012; Verlhac et al., 2015; Minowa-Nozawa et al., 2017); thus, we sought to ascertain whether Tollip also participates in galectin recruitment. EmGFP-galectin-3 and -8 colocalized with GAS-containing LC3-vacuoles at a similar rate in wild-type and Tollip-knockout cells, whereas the recruitment of EmGFP-galectin-7 was significantly decreased in Tollip-knockout cells (**Figures 4A, B**). Moreover, the recruitment of endogenous galectin-1 to LC3-vacuoles was disrupted and significantly declined in Tollip-knockout cells (**Figures 4C, D**). To determine whether Tollip knockout also impacts the efficiency of GAS invasion into the cytosol, immunostaining for SLO was carried out (Lin et al., 2019), which demonstrated that the cytosolic invasion of GAS was not influenced by Tollip knockout (**Figures 4E, F**). Furthermore, immunoprecipitation results showed that EmGFP-galectin-7 coprecipitated with FLAG-Tollip (**Figure 4G**), which indicated that Tollip interacts with galectin-7. These results implied that Tollip functions in bacterial autophagy by regulating the recruitment of galectin-1 and -7.

### Galectin-1 and -7 Participate in Xenophagic Defense against Group A *Streptococcus* Infection

**Figure 5:**
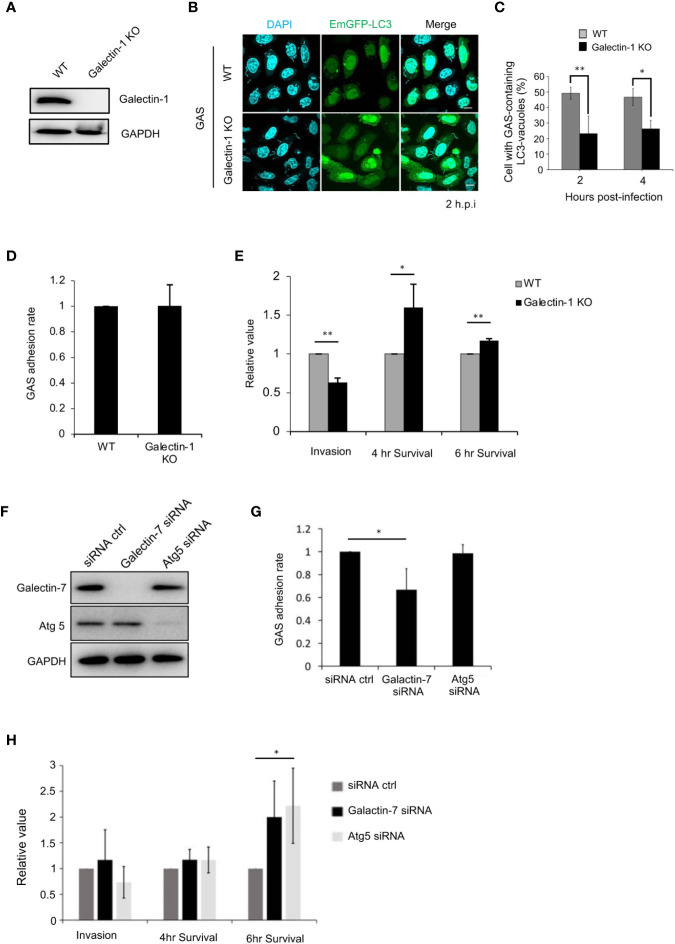
Galectin-1 and -7 play critical roles in the defense against GAS infection. **(A)** Galectin-1-knockout HeLa cells were generated using the CRISPR/Cas9 gene-editing system. Immunoblotting was used to detect galectin-1 protein expression in wild-type and galectin-1-knockout cells. **(B)** Wild-type and galectin-1-knockout HeLa cells were transfected with EmGFP-LC3; after infecting with GAS for 2 or 4 h, immunofluorescence analysis was used to examine the formation of bacterium-containing LC3-vacuoles. **(C)** Cells harboring GAS-containing LC3-vacuoles were manually counted using immunofluorescence confocal microscopy. In each experiment, >100 autophagosomes were evaluated per sample; bars: means ± SD of 3 independent experiments. **(D)** Adhesion rate of GAS in wild-type and galectin-1-knockout HeLa cells. Bars: means ± SD of 3 independent experiments. **(E)** Invasion rate and survival rate of GAS in wild-type and galectin-1-knockout HeLa cells. **(F)** Galectin-7- or Atg5-knockdown HaCaT cells were generated using siRNAs. Immunoblotting was used to detect galectin-7 and Atg5 protein expression. **(G)** Adhesion rate of GAS in wild-type and galectin-7- or Atg5-knockdown HaCaT cells. Bars: means ± SD of 3 independent experiments. **(H)** Invasion rate and survival rate of GAS in wild-type and galectin-7- or Atg5-knockdown HaCaT cells. Bars: means ± SD of 3 independent experiments. *P < 0.02, **P < 0.01.

To elucidate the role of galectin-1 in bacterial autophagy, galectin-1-knockout HeLa cells were constructed (**Figure 5A**). In these knockout cells, the formation of GAS-containing LC3-vacuoles was found to be impeded (**Figures 5B, C**). The GAS adhesion rate in galectin-1-knockout cells did not differ relative to the rate observed in the control (**Figure 5D**), however, the invasion rate was significantly decreased in the knockout cells (**Figure 5E**). Furthermore, galectin-1 knockout resulted in a significant rise in the proliferation rate of intracellular GAS at 4 and 6 h post-infection (**Figure 5E**).

Lastly, we examined the role of galectin-7 during GAS infection. In HeLa cells, galectin-7 protein was detected only weakly (**Figure S4A**); hence, galectin-7 expression was knocked down in HaCaT cells using siRNAs (**Figure 5F**). Our analysis here first revealed that galectin-7 was colocalized with galectin-3 and LC3 during GAS infection (**Figures S4B, C**), which indicated that galectin-7 participates in bacterial autophagy in HaCaT cells. Next, galectin-7 function during bacterial infection was investigated by performing infection assays on galectin-7-knockdown cells. In these knockdown cells, the GAS adhesion rate was lower than that seen in control cells (**Figure 5G**) but the GAS invasion rate was not decreased; however, the GAS survival rate was significantly increased. There was a similar increase in the survival rate in autophagy-deficient Atg5-knockdown cells (**Figure 5H**). Taken together, these results signified that galectin-1 and -7 respond to GAS infection and function in bacterial autophagy to defend against bacterial infection.

## Discussion

**Figure 6:**
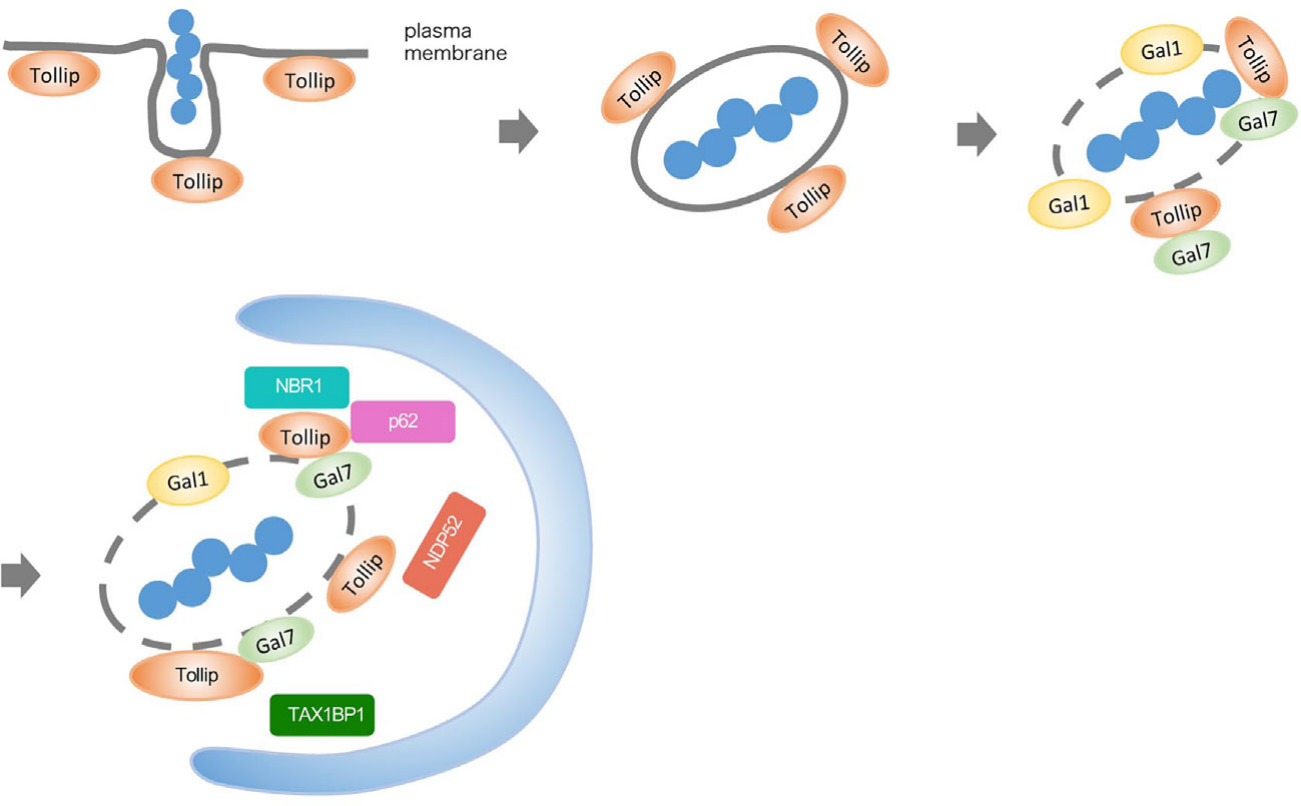
Model depicting Tollip function in xenophagy. Tollip localizes on the inner surface of the cell membrane by binding to PtdIns*3P* through its C2 domain. After GAS invasion through endocytosis, Tollip surrounds GAS before GAS damages the endosomal membrane and escapes into the cytosol. After GAS damages the endosomal membrane, galectin-1 and -7 are recruited to the membrane fragments and associate with Tollip, and then other autophagy receptors, including NBR1, TAX1BP1, p62, and NDP52, are recruited.

Tollip has been reported to function as an autophagy receptor and to be involved in the clearance of protein aggregates (Lu et al., 2014). However, in the present study, we identified a previously unknown function of Tollip. We determined that Tollip participates in bacterial autophagy in response to bacterial infection by regulating the recruitment of other autophagy receptors and galectins, and we further demonstrated that galectin-1 and -7 play a crucial role in the defense against bacterial infection (**Figure 6**).

Tollip is targeted to the membrane through PtdIns*3P* binding, is involved in early endosome trafficking (Ankem et al., 2011; Xiao et al., 2015), and is confined around bacteria before endomembrane damage; therefore, Tollip has been proposed to function as an endosome-resident autophagy receptor. Considering these findings, we speculate that certain molecules that localize on the inner cell membrane may play a critical role in autophagosome formation and function in the first line of response to bacterial infection. We previously reported that endosome-resident RAB35 and TBC1D9 recruit NDP52 for triggering xenophagy (Minowa-Nozawa et al., 2017; Nozawa et al., 2020), and thus a highly sophisticated mechanism may exist for regulating autophagy receptors in response to infection. Intriguingly, our results showed that incomplete LC3-positive vacuoles (perhaps forming autophagic vacuoles) were accumulated in Tollip-knockout cells but not in cells in which galectin-1 or -7 was depleted; it is therefore inferred that Tollip potentially plays an additional (as yet unknown) role in membrane elongation that is not mediated by galectins. Further studies are warranted to identify and comprehensively clarify the function(s) of Tollip in autophagosome formation.

Autophagy receptors have been demonstrated to be involved in autophagosome formation (Birgisdottir et al., 2013); thus, as per our expectation, the formation of bacterium-containing autophagosomes and autophagolysosomes was impaired following Tollip knockout. Tollip knockout also impeded the recruitment of other autophagy receptors, including NBR1, TAX1BP1, and NDP52, to GAS-containing autophagosomes, which implies that Tollip functions as a linker between autophagy receptors and GAS-containing vacuoles. NDP52 is required for autophagosome formation and TAX1BP1 is involved in autophagosome -lysosome fusion during GAS infection; therefore, the inhibition of autophagosome and autophagolysosome formation following Tollip knockout may result from defective recruitment of these autophagy receptors. NBR1 was also recruited to GAS-containing autophagosomes in a Tollip-dependent manner, but the roles of NBR1 in xenophagy are currently unknown.

In Tollip-knockout cells, the recruitment of galectin-1 and -7 was also disrupted. However, cells harboring SLO-labeled GAS were not decreased among Tollip-knockout cells, and Tollip knockout did not affect galectin-3 and -8 targeting of GAS; this indicates that Tollip knockout did not perturb GAS internalization into the cytosol. Moreover, although galectins are considered to target damaged endosomal membranes by recognizing intracellularly exposed sugar chains (Thurston et al., 2012; Jia et al., 2018), our results showed that the exposed sugar chains as well as the endosomal proteins facilitate the recruitment of specific galectins (galectin-1 and -7) to bacterially damaged endosomes.

Galectins function as sensors that respond to endomembrane damage during bacterial escape into the cytosol (Paz et al., 2010). Galectin-3, -8, and -9 have been reported to be recruited to damaged bacterium-containing vacuoles and to participate in the defense against bacterial infection (Paz et al., 2010; Thurston et al., 2012; Chauhan et al., 2016). Herein, we found that galectin-1 and -7 also play a critical role in the intracellular immune response against bacterial infection. Intracellular galectin-1 was previously reported to be not accumulated on damaged vacuoles containing *Salmonella enterica* serovar Typhimurium, *Listeria monocytogenes*, or *Shigella flexneri* (Thurston et al., 2012); however, we observed that intracellular galectin-1 was accumulated on *S. aureus*- and GAS-containing damaged vacuoles. Thus, intracellular galectins appear to exhibit distinct recruitment preferences for different bacterial species; consequently, bacterial species is a key factor in triggering the recruitment of distinct intracellular galectins. Our results demonstrated that galectin-1 and -7 play a crucial role in the bacterial-autophagy defense against GAS infection.

Intracellular galectin-7 is recognized as a multifunctional protein involved in processes such as cell proliferation, apoptosis, and cell migration (Bernerd et al., 1999; Gendronneau et al., 2008), and galectin-7 also serves as a marker of cancer metastasis (Moisan et al., 2003). Although a previous study reported that galectin-7 is not expressed in HeLa cells (Kuwabara et al., 2002), our immunoblotting analysis revealed a weak band indicating low-level galectin-7 expression in HeLa cells. Nevertheless, our examination of HaCaT cells revealed that intracellular galectin-7 clearly colocalized with and surrounded LC3-positive GAS, which indicates that intracellular galectin-7 is involved in bacterial autophagy. Thus, the decline in the GAS adhesion rate observed in galectin-7-knockdown cells may be explained by the fact that galectin-7 depletion resulted in a disruption of the E-cadherin dynamics that is required for bacterial adhesion to host-cell surface (Anderton et al., 2007; Advedissian et al., 2017). Notably, GAS proliferation was increased following galectin-7 knockdown in HaCaT cells, which indicates that intracellular galectin-7 plays a critical role in intracellular immunity in the response against bacterial infection.

In summary, the present study revealed that Tollip functions as a bacterial-autophagy receptor to defend against bacterial infection. Furthermore, our study demonstrated that galectin-1 and -7 are involved in bacterial autophagy and play a critical role in cellular defense against GAS infection.

## Data Availability Statement

The original contributions presented in the study are included in the article/**Supplementary Material** further inquiries can be directed to the corresponding author.

## Author Contributions

C-YL performed the experiments, analyzed the data, and drafted the manuscript. TN, AM-N, HT, MH, and JI assisted with data collection. C-YL, TN, and IN conceived the study, provided reagents, and revised the paper. All authors contributed to the article and approved the submitted version.

## Funding

The authors acknowledge financial support from Grants-in-Aid for Scientific Research (16H05188, 15K15130, 26462776, 17K19552), the Takeda Science Foundation, the Research Program on Emerging and Re-emerging Infectious Diseases (18fk0108044h0202, 18fk0108073h0001), and J-PRIDE (18fm0208030h0002) from Japan Agency for Medical Research and Development, AMED.

## Conflict of Interest

The authors declare that the research was conducted in the absence of any commercial or financial relationships that could be construed as a potential conflict of interest.

## Supplementary Material

Supplementary Figure 1 Colocalization of Tollip and galectins. **(A)** HeLa cells were infected with GAS for 4 h, fixed and stained with anti-GAC antibody (magenta) and DAPI (cyan). Scale bar, 10 μm. **(B)** HeLa cells co-transfected with mCherry-Tollip and EmGFP-galectins were infected with GAS. SLO protein from GAS was labeled with mouse monoclonal anti-streptolysin antibody. Immunofluorescence analysis was performed to examine the localization of EmGFP-galectins, mCherry-Tollip, and SLO at 4 hpi. **(C)** Tollip-positive and EmGFP-galectin-positive puncta among SLO-positive puncta were manually counted using immunofluorescence confocal microscopy; >100 SLO puncta were evaluated per sample.

Supplementary Figure 2 mCherry-Tollip and galectin-1 and -7 were recruited to *S. aureus*. **(A)** HeLa cells co-transfected with mCherry-Tollip and EmGFP-galectin-1 were infected with *S. aureus*; at 4 hpi, immunofluorescence analysis was performed to examine the localization of EmGFP-galectin-1 and mCherry-Tollip. Insets: enlarged boxed areas. All images are representative of at least 3 independent experiments. Scale bar, 10 μm. **(B)** HeLa cells co-transfected with mCherry-LC3 and EmGFP-galectin-7 were infected with *S. aureus*. Immunofluorescence analysis was used to examine the localization of EmGFP-galectin-7 and mCherry-LC3 at 4 hpi. Insets: enlarged boxed areas. All images are representative of at least 3 independent experiments. Scale bar, 10 μm.

Supplementary Figure 3 LC3-positive autophagosomes surrounding GAS were incomplete and not closed in Tollip-knockout cells. Wild-type and Tollip-knockout HeLa cells transfected with EmGFP-LC3 were infected with GAS, and immunofluorescence analysis was performed at 4 hpi to examine the formation of LC3-positive autophagosomes. All images are representative of at least 3 independent experiments. Scale bar, 10 μm.

Supplementary Figure 4 Galectin-7 was recruited to GAS and colocalized with mCherry-LC3 and galectin-3. **(A)** Immunoblotting was performed to detect galectin-7 protein in A549, Ca9-22, HeLa, and HaCaT cells. **(B)** HaCaT cells were infected with GAS, and at 4 hpi, endogenous galectin-7 and -3 were labeled with antibodies and the localization of the proteins was examined using immunofluorescence microscopy. Insets: enlarged boxed areas. All images are representative of at least 3 independent experiments. Scale bar, 10 μm. **(C)** HaCaT cells transfected with mCherry-LC3 were infected with GAS, and at 4 hpi, endogenous galectin-7 was labeled with an antibody and immunofluorescence analysis was used to examine the localization of mCherry-LC3 and galectin-7. Insets: enlarged boxed areas. All images are representative of at least 3 independent experiments. Scale bar, 10 μm.
