# Supplementary figures and images for: Autophagy Receptor Tollip Facilitates Bacterial Autophagy by Recruiting Galectin-7 in Response to Group A Streptococcus Infection
Source: Front Cell Infect Microbiol. 2020 Dec 23;10:583137. doi: 10.3389/fcimb.2020.583137 (PMC7786282; doi:10.3389/fcimb.2020.583137)

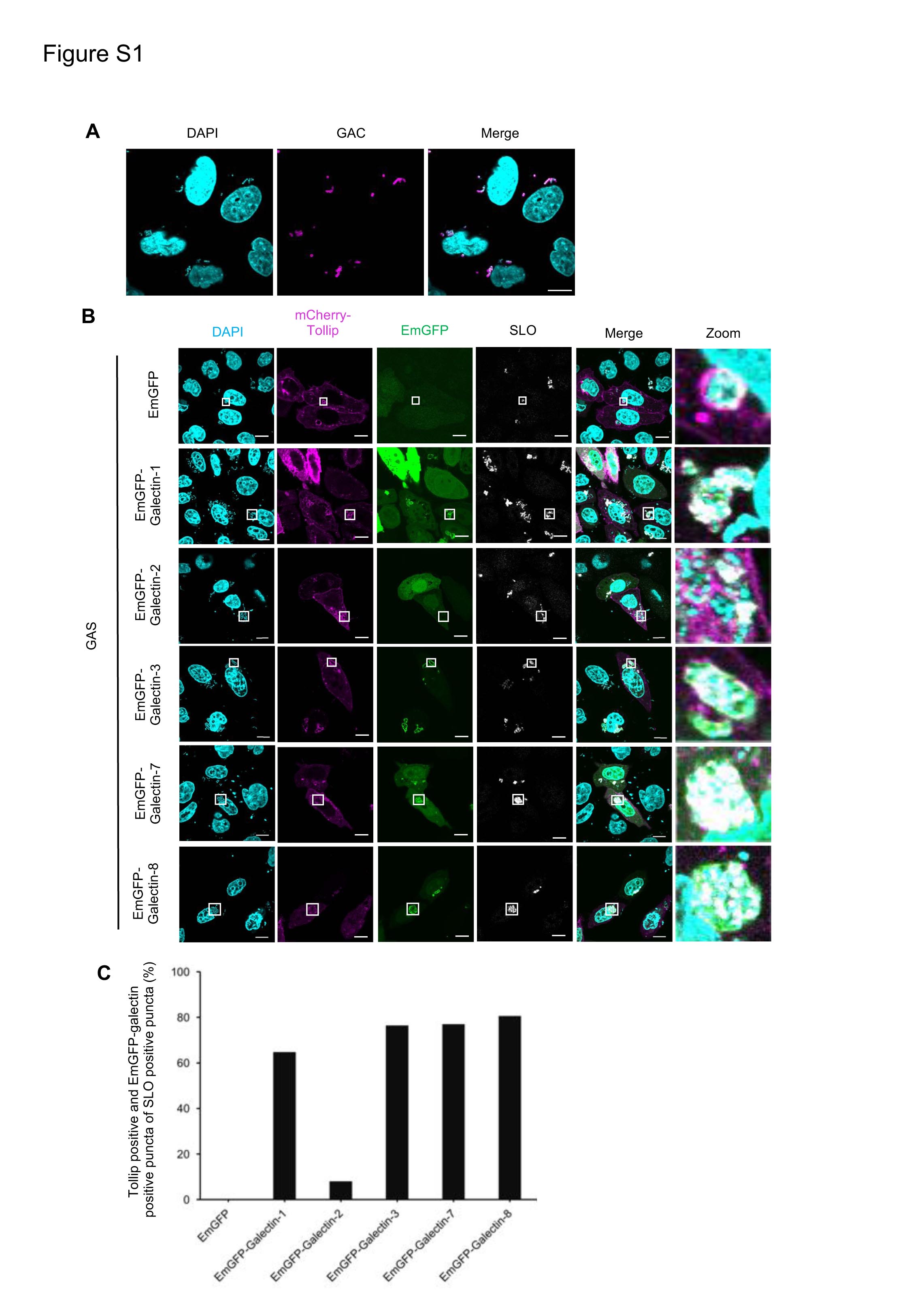

Supplement: Supplementary Figure 1 — Colocalization of Tollip and galectins. (A) HeLa cells were infected with GAS for 4 h, fixed and stained with anti-GAC antibody (magenta) and DAPI (cyan). Scale bar, 10 μm. (B) HeLa cells co-transfected with mCherry-Tollip and EmGFP-galectins were infected with GAS. SLO protein from GAS was labeled with mouse monoclonal anti-streptolysin antibody. Immunofluorescence analysis was performed to examine the localization of EmGFP-galectins, mCherry-Tollip, and SLO at 4 hpi. (C) Tollip-positive and EmGFP-galectin-positive puncta among SLO-positive puncta were manually counted using immunofluorescence confocal microscopy; >100 SLO puncta were evaluated per sample. [file Image_1.jpeg]

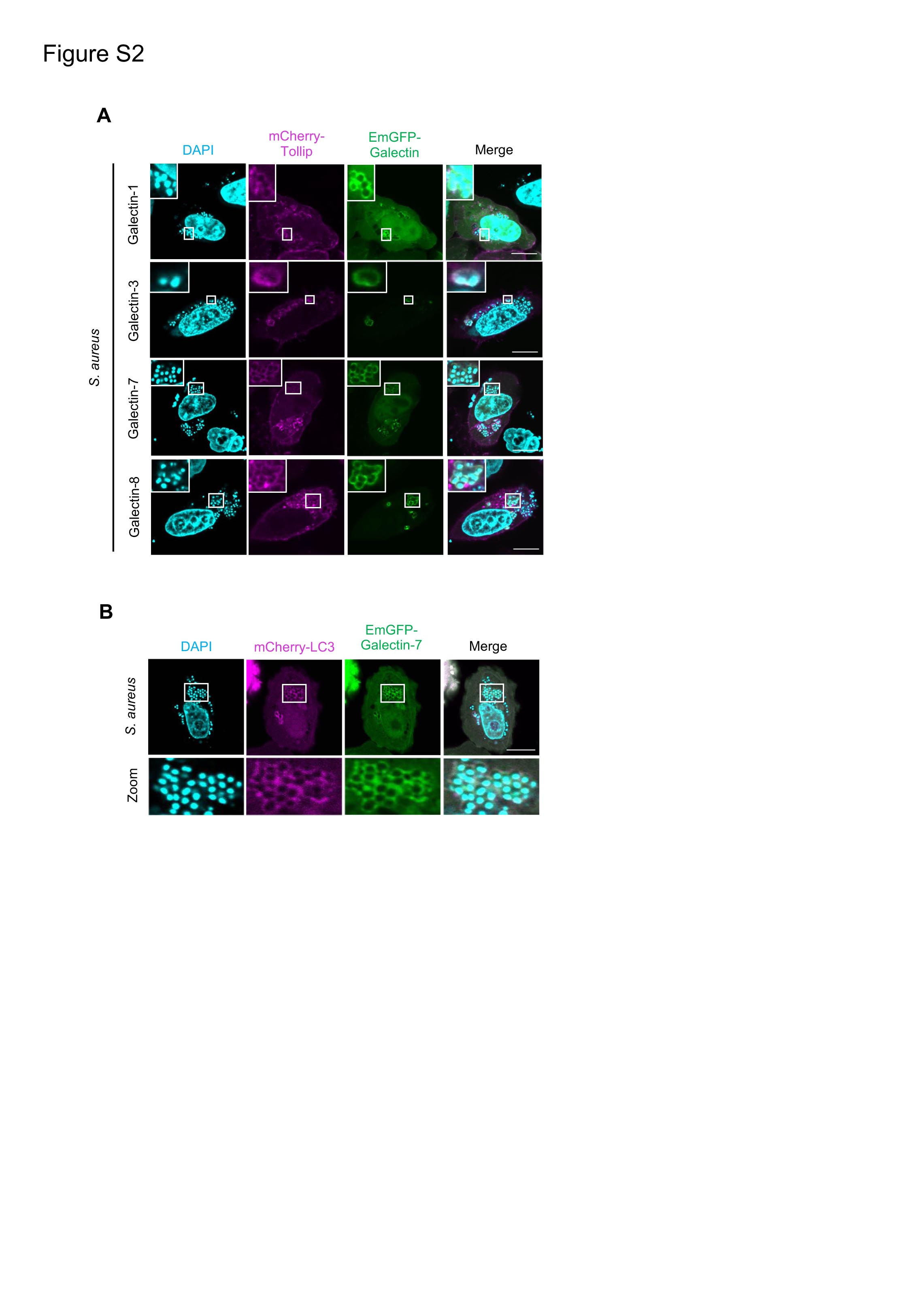

Supplement: Supplementary Figure 2 — mCherry-Tollip and galectin-1 and -7 were recruited to S. aureus. (A) HeLa cells co-transfected with mCherry-Tollip and EmGFP-galectin-1 were infected with S. aureus; at 4 hpi, immunofluorescence analysis was performed to examine the localization of EmGFP-galectin-1 and mCherry-Tollip. Insets: enlarged boxed areas. All images are representative of at least 3 independent experiments. Scale bar, 10 μm. (B) HeLa cells co-transfected with mCherry-LC3 and EmGFP-galectin-7 were infected with S. aureus. Immunofluorescence analysis was used to examine the localization of EmGFP-galectin-7 and mCherry-LC3 at 4 hpi. Insets: enlarged boxed areas. All images are representative of at least 3 independent experiments. Scale bar, 10 μm. [file Image_2.jpeg]

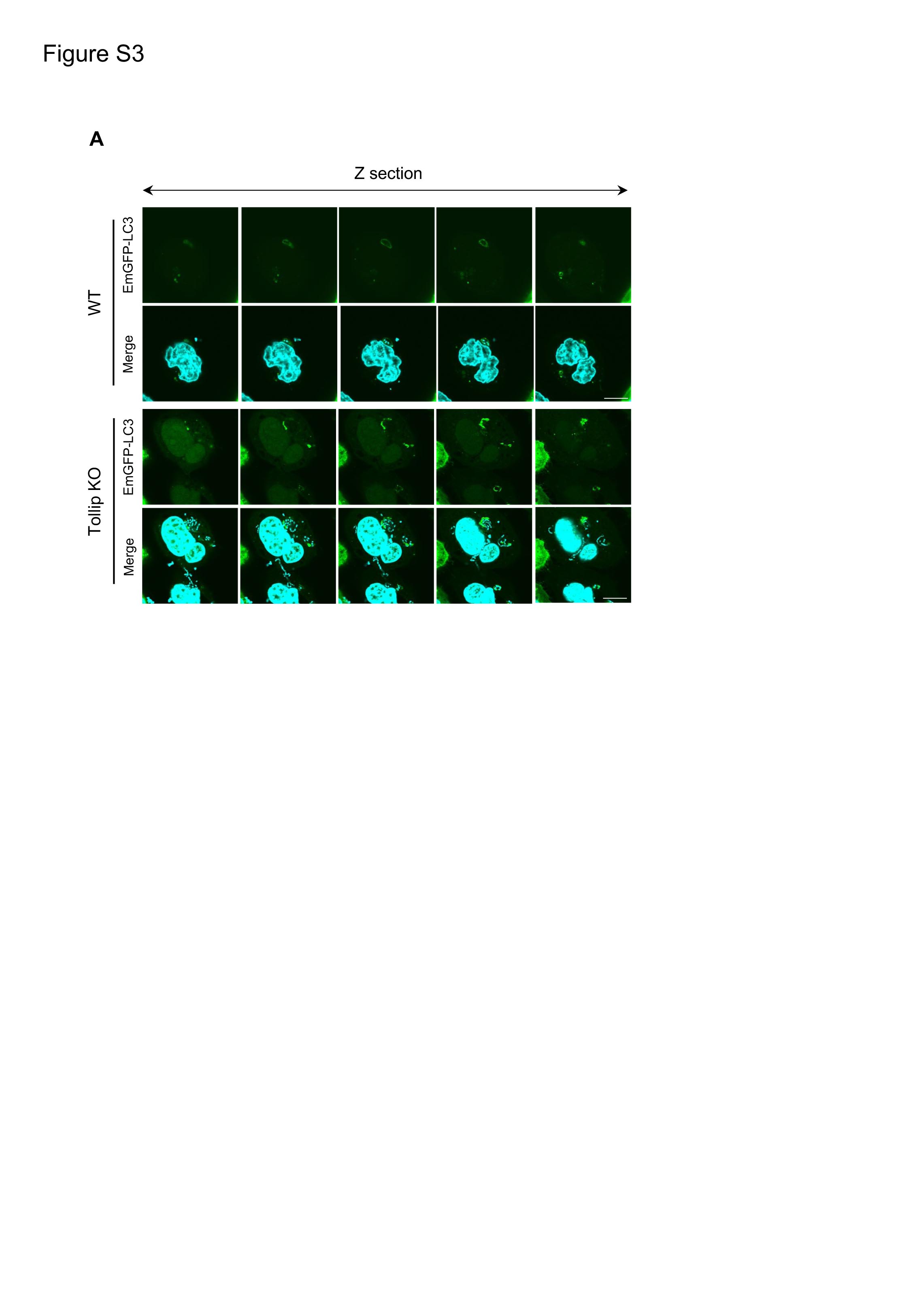

Supplement: Supplementary Figure 3 — LC3-positive autophagosomes surrounding GAS were incomplete and not closed in Tollip-knockout cells. Wild-type and Tollip-knockout HeLa cells transfected with EmGFP-LC3 were infected with GAS, and immunofluorescence analysis was performed at 4 hpi to examine the formation of LC3-positive autophagosomes. All images are representative of at least 3 independent experiments. Scale bar, 10 μm. [file Image_3.jpeg]

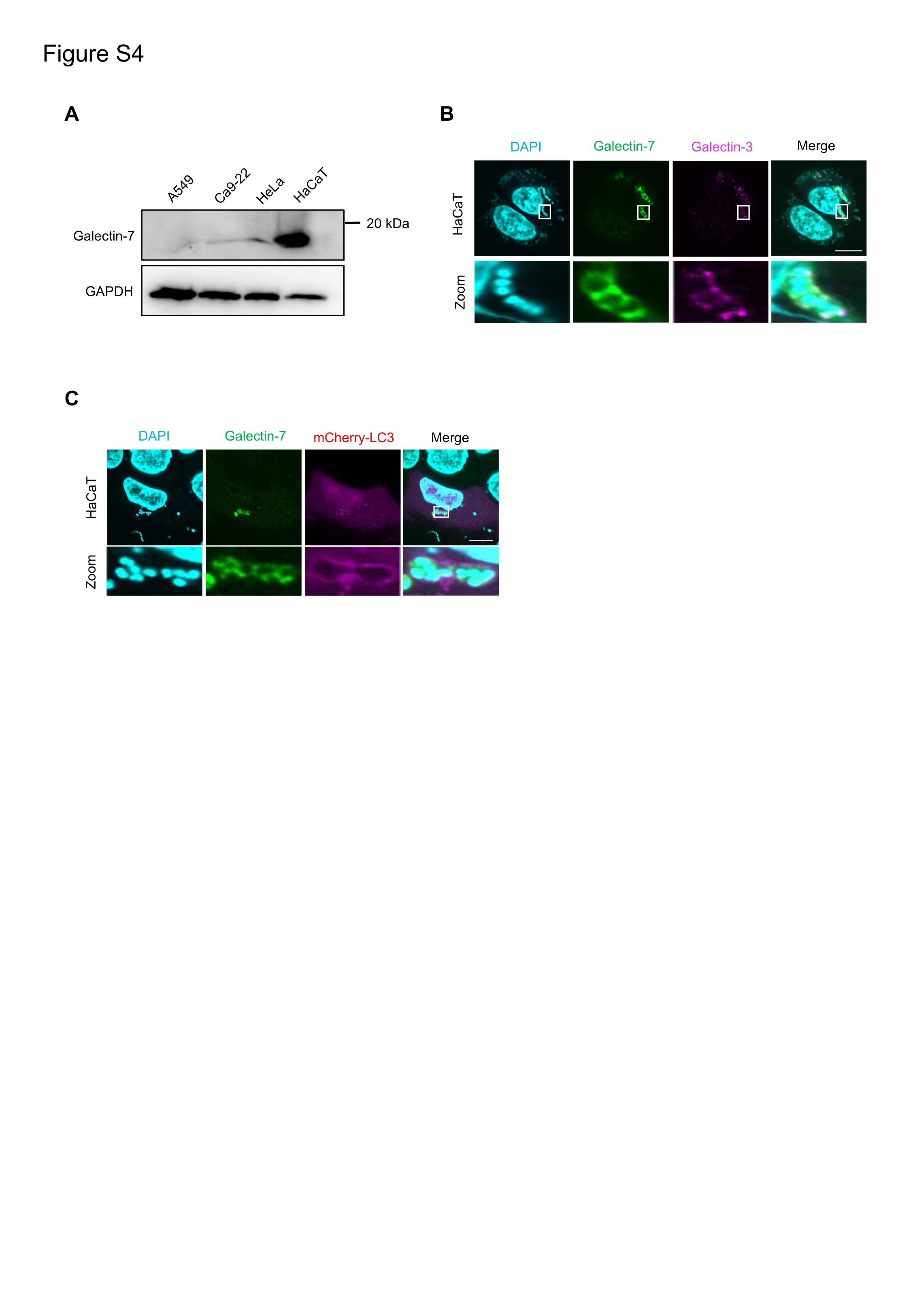

Supplement: Supplementary Figure 4 — Galectin-7 was recruited to GAS and colocalized with mCherry-LC3 and galectin-3. (A) Immunoblotting was performed to detect galectin-7 protein in A549, Ca9-22, HeLa, and HaCaT cells. (B) HaCaT cells were infected with GAS, and at 4 hpi, endogenous galectin-7 and -3 were labeled with antibodies and the localization of the proteins was examined using immunofluorescence microscopy. Insets: enlarged boxed areas. All images are representative of at least 3 independent experiments. Scale bar, 10 μm. (C) HaCaT cells transfected with mCherry-LC3 were infected with GAS, and at 4 hpi, endogenous galectin-7 was labeled with an antibody and immunofluorescence analysis was used to examine the localization of mCherry-LC3 and galectin-7. Insets: enlarged boxed areas. All images are representative of at least 3 independent experiments. Scale bar, 10 μm. [file Image_4.jpeg]
